# Density and maturity of peritumoral tertiary lymphoid structures in oesophageal squamous cell carcinoma predicts patient survival and response to immune checkpoint inhibitors

**DOI:** 10.1038/s41416-023-02235-9

**Published:** 2023-04-04

**Authors:** Yoshinori Hayashi, Tomoki Makino, Eiichi Sato, Kenji Ohshima, Yuya Nogi, Takashi Kanemura, Keiichiro Honma, Kotaro Yamashita, Takuro Saito, Koji Tanaka, Kazuyoshi Yamamoto, Tsuyoshi Takahashi, Yukinori Kurokawa, Hiroshi Miyata, Kiyokazu Nakajima, Hisashi Wada, Eiichi Morii, Hidetoshi Eguchi, Yuichiro Doki

**Affiliations:** 1 Department of Gastroenterological Surgery, Graduate School of Medicine, Osaka University, Osaka, Japan; 2 Department of Clinical Research in Tumor Immunology, Graduate School of Medicine, Osaka University, Osaka, Japan; 3 Department of Pathology, Institute of Medical Science (Medical Research Center), Tokyo Medical University, Tokyo, Japan; 4 Department of Pathology, Graduate School of Medicine, Osaka University, Osaka, Japan; 5 Department of Gastroenterological Surgery, Osaka International Cancer Institute, Osaka, Japan; 6 Department of Pathology, Osaka International Cancer Institute, Osaka, Japan

**Keywords:** Oesophageal cancer, Immunoediting, Surgical oncology, Oesophageal cancer, Cancer microenvironment

## Abstract

**Background:**

Tertiary lymphoid structures (TLSs) are ectopic lymphoid aggregates in non-lymphoid tissues, which are associated with improved prognosis in some cancer types. This study aimed to investigate the clinical significance of TLSs in oesophageal cancer (EC).

**Methods:**

In a series of 316 EC surgical specimens from two different institutes, we evaluated the density and maturity of peritumoral TLSs using haematoxylin/eosin, immunohistochemistry, and multiplex immunofluorescence staining. We analysed the association between TLSs and clinicopathological parameters. The clinical significance of TLSs was further evaluated in a different cohort of 34 patients with recurrent EC treated with anti-PD-1 antibody.

**Results:**

Tumours with high TLS density predominantly consisted of matured TLSs. High TLS density was significantly associated with less advanced tumour stage, absence of lymphatic/vascular invasion, better serum nutrition parameters (neutrophils count, albumin, neutrophil-to-lymphocyte ratio, and prognostic nutritional index), and prolonged survival. This survival trend was more remarkable in cases with matured TLSs, which represented an increased population of CD138^+^ plasma cells. In the second EC cohort, TLS density predicted the clinical response to anti-PD-1 antibody and patient survival.

**Conclusion:**

The density and maturity of peritumoral TLSs are useful parameters for predicting long-term survival and response to anti-PD-1 antibody treatment in EC patients.

## Introduction

Oesophageal cancer (EC) is a common malignant gastrointestinal disease, and the sixth leading cause of cancer-related death worldwide [1]. Despite the development of multimodal treatments, including combinations of surgery, chemotherapy, and radiotherapy [2–6], EC still has an unsatisfactory prognosis. The limited efficacy of conventional treatment strategies has prompted the development of novel treatments for EC, including with immune checkpoint inhibitors (ICI) [7, 8].

The recent development of ICI for several cancer types highlights the importance of the tumour immune microenvironment [9, 10]. Research has demonstrated that immune cells infiltrating the tumour microenvironment (TME) play important roles in the antitumor responses and clinical outcomes. Notably, tumour-specific T lymphocytes, known as tumour-infiltrating T lymphocytes (TILs), are reportedly associated with better prognosis in several cancer types [11–13]. We previously demonstrated that TILs have clinical impacts on treatment efficacy and survival in EC patients [9]. On the other hand, the clinical significance of other infiltrates in the TME of EC remains unclear.

Tertiary lymphoid structures (TLSs) are ectopic lymphoid formations that develop in non-lymphoid tissue with chronic inflammatory disorders [14, 15]. They are also expressed in cancers, particularly inflammatory-related cancers, as structured aggregates of immune cells, which show an organisation similar to secondary lymphoid organs [16, 17]. They are considered a possible facilitator of the influx of immune cells into the tumour site, and have attracted interest as a potential means of improving antitumor immunity. Previous studies show that the presence and maturity of TLSs are correlated with tumour progression or prognosis, and can predict responses to chemotherapy or immunotherapy in several tumours [18–22]. However, these relationships have not been studied in EC.

In this study, we aimed to assess the expression and maturity of peritumoral TLSs in EC, by performing hematoxylin/eosin (H&E) staining and immunohistochemistry (IHC). We also analysed the cell profile of TLSs by multiplex immunofluorescence (IF) staining.

## Methods

### Patients and samples

This study enrolled a total of 316 consecutive patients with oesophageal squamous cell carcinoma who underwent esophagectomy at two institutions, Osaka University Hospital and Osaka International Cancer Institute, from January 2001 to December 2017. All patients underwent curative surgical resection without preoperative chemotherapy or (chemo-) radiotherapy. This study also included a different cohort of 34 EC patients who received anti-PD-1 antibody as a second- or later-line treatment for postoperative recurrence at Osaka University Hospital from June 2014 to September 2021. All participants gave informed consent, and the study procedures were approved by the Institutional Review Board of Osaka University Hospital (No. 20056).

The patients’ clinicopathological information was collected from the clinical database and pathological reports of each institution. Haematological findings and indices were based on preoperative data. As serum nutrition parameters, the neutrophil-to-lymphocyte ratio (NLR) and prognostic nutrition index (PNI) were calculated as previously described: NLR = total neutrophil count (/mm^3^)/total lymphocyte count (/mm^3^); PNI = 10 × serum albumin (g/dL) + 0.005 × total lymphocyte count (/mm^3^) [23, 24]. Tumour staging was performed according to the eighth edition of the Union for International Cancer Control TNM classification system [25]. The standard nivolumab regimen was intravenous administration of 240 mg nivolumab, every 14 days [7]. Responses to ICI therapy were assessed as best overall response according to Response Evaluation Criteria in Solid Tumours (RECIST), version 1.1 [26].

Formalin-fixed paraffin-embedded surgical specimens were collected from all patients, and serial 4-µm-thick sections were prepared for H&E, IHC, and multiplex IF staining [27–29]. The sample blocks containing the deepest portion of tumour tissue were used.

### Immunohistochemistry

All slides were manually processed. Sections were deparaffinized in xylene, placed in citric acid-based retrieval buffer (pH 6.0), and heated at 110 °C for 15 min in a pressure cooker, according to the manufacturer’s protocol. The slides were washed in distilled water, treated with 3% H_2_O_2_ for 20 min, washed three times in 0.1% Triton X-100/PBS, and then blocked using 1.5% serum solution/PBS. Antibodies were diluted in PBS. Samples were incubated with primary antibodies overnight at 4 °C, washed three times, incubated with secondary antibodies for 20 min at room temperature, and then washed three times. Detection was performed using an avidin-biotinylated enzyme complex kit (Vectastain ABC Kit, PK6100, Vector, Newark, USA) and 3ʹ-3-diaminobenzidine (DAB) substrate (Wako, Japan), following the manufacturer’s protocol. Slides were counterstained with hematoxylin, washed, dehydrated with ethanol, and mounted. The primary antibodies were CD21 (2G9, NCL-L-CD21-2G9; Leica Biosystems, Germany), CD23 (SP23, ab16702; Abcam, Cambridge, UK), PD-1 (NAT105, ab52587; Abcam, Cambridge, UK) and the secondary antibodies were horse anti-mouse IgG (BA-2000; Vector, Newark, USA) and goat anti-rabbit IgG (BA-1000; Vector, Newark, USA). The serum-blocking solutions were horse (S-2000; Vector, Newark, USA) and goat (S-1000; Vector, Newark, USA).

### Pathological evaluation of TLS expression and maturity

To assess TLS expression, we identified dense lymphatic aggregates in the whole tumour area and counted their numbers in H&E-stained sections. For quantitative evaluation, we scanned whole slides, and regarded the peritumoral region as the range within 1000 µm from the boundary of the tumour nest—which is where TLSs were exclusively formed in our preliminary experiment. The area of each peritumoral region was measured using a digital microscopy system (BZ-X710; Keyence, Osaka, Japan) and its dedicated analytical software (BZ-H3A; Keyence, Osaka, Japan). We defined TLS density by calculating the number of TLSs per mm^2^ in the peritumoral region, as the standardisation of pathological expression.

To assess TLS maturity, serial sections were stained with H&E and IHC for CD21 and CD23 (markers of follicular dendritic cells and germinal centres, respectively). Samples from each case were evaluated based on both the morphological features and the combination of molecular signals, according to the previously reported methods [21, 30]. We classified each TLS into three maturation categories, as follows: early TLSs (E-TLSs), vague dense lymphocytic aggregations lacking CD21 or CD23 signals; primary follicle-like TLSs (PFL-TLSs), definite round- or oval-shaped clusters of small lymphocytes with CD21 signals but not CD23 signals; secondary follicle-like TLSs (SFL-TLSs), follicles with a definite germinal centre formation comprising large lymphocytes with clear cytoplasm, and having both CD21 and CD23 signals. For each case, we defined the density and proportion of each maturation category.

### Multiplex immunofluorescence staining

The experimental process of specimen preparation was the same as for a single IHC. Primary antigen retrieval was performed using High pH Target Retrieval Solution (DAKO, Glostrup, Denmark). The primary antibodies were anti-CD1c (mouse monoclonal, clone OTI2F4; Abcam, Cambridge, UK), anti-CD4 (mouse monoclonal, clone 4B12; Invitrogen, Waltham, USA), anti-CD8 (mouse monoclonal, clone C8/144b; DAKO, Glostrup, Denmark), anti-CD19 (mouse monoclonal, clone LE-CD19; Invitrogen, Waltham, USA), anti-CD21 (rabbit monoclonal, clone SP32; Abcam, Cambridge, UK), and anti-CD138 (mouse monoclonal, clone MI15; DAKO, Glostrup, Denmark). For secondary detection, we used a horseradish peroxidase-labelled detection system (EnVision plus; DAKO, Glostrup, Denmark) as a catalyst for fluorophore-conjugated tyramide. Antigen stripping was performed using Immunoactive Retrieval Buffer (pH 6; Matsunami Glass, Osaka, Japan). Tyramide signal amplification was performed using Opal fluorophore reagents (Akoya Biosciences, Marlboro, USA); Opal 520, Opal 540, Opal 570, Opal 620, Opal 650, and Opal 690 were used for CD1c, CD4, CD8, CD19, CD21, and CD138, respectively. DAPI counterstaining was performed using Spectral DAPI solution (Akoya Biosciences, Marlboro, USA).

### Quantitative imaging analyses

For each TLS, co-localised signals were detected, and tissue images (669 × 500 µm in size) were captured into multispectral images using an automated pathology imaging system (Vectra 3.0; Perkin Elmer, Waltham, USA). The number of fluorescent-positive cells was counted using automated analytical software (InForm; Perkin Elmer, Waltham, USA), with the appropriate programmed cell-count algorithm [10]. We determined the expression pattern of CD1c^+^ dendritic cells, CD4^+^ T lymphocytes, CD8^+^ T lymphocytes, CD19^+^ B lymphocytes, CD21^+^ follicular dendritic cells, and CD138^+^ plasma cells. We acquired all the cell-count data with any molecular signals, and calculated both the density and proportion of each signal in a field for quantitative analysis.

### Statistical analysis

We analysed relationships between clinicopathological characteristics using the chi-square test or Fisher’s exact test for categorical variables. Continuous variables are expressed as median and range, and were analysed using the Mann–Whitney *U* test. For survival analysis, the Kaplan–Meier method was used to evaluate the survival time distribution, the log-rank test for comparisons, and a Cox proportional hazard model for computing the hazard ratio (HR) and univariate/multivariate analysis. The prognostic variables that showed a significant association in univariate analysis were included in multivariate analysis. *P* < 0.05 was considered to indicate statistical significance. All *P* values were two-sided. Statistical analyses and data descriptions were performed using JMP Pro software (version 16.2.0; SAS Institute Inc., Cary, USA).

## Results

### The expression and maturity of tumour-associated TLSs in EC

**Fig. 1:**
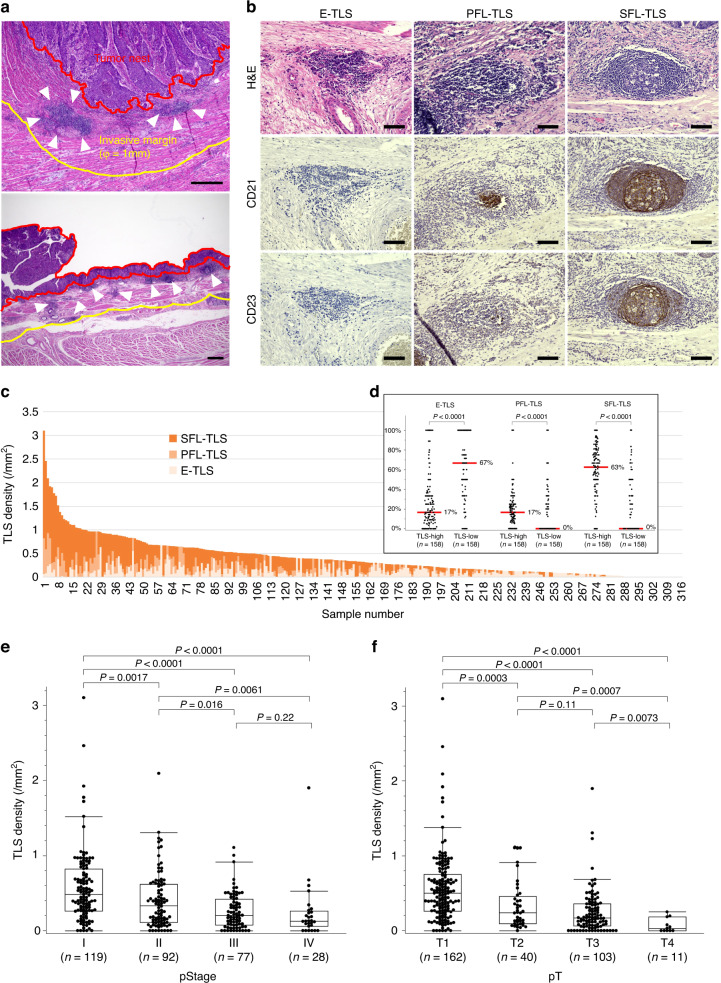
Evaluation of peritumoral tertiary lymphoid structure (TLS) according to density and maturity by hematoxylin/eosin (H&E) staining and immunohistochemistry (IHC) of CD21 and CD23. **a** Representative sections for counting peritumoral TLSs (arrowheads) within peritumoral regions. **b** Representative images of TLSs in each maturation category as evaluated by H&E and IHC. Early TLSs (E-TLSs) are vague dense lymphocytic aggregations without CD21 or CD23 signals. Primary follicle-like TLSs (PFL-TLSs) are definite round- or oval-shaped clusters of small lymphocytes with CD21 signals, but no CD23 signals. Secondary follicle-like TLSs (SFL-TLSs) are follicles with a definite germinal centre formation, comprising large lymphocytes with clear cytoplasm, and having both CD21 and CD23 signals. **c** Distribution of TLS density and proportion of each TLS maturation category among 316 oesophageal cancer patients. **d** Proportion of TLS maturity levels according to TLS density. Red lines and the numbers next to them show median values. **e, f** TLS density according to pT (**e**) and pStage (**f**). Scale bars: **a** = 500 µm; **b** = 100 µm. Comparisons were made by Mann–Whitney *U* test.

Evaluation of the presence and localisation of tumour-associated TLSs in EC revealed that most TLSs were formed in peritumoral regions, and there were no dense lymphocytic aggregates in the intratumoral regions (Fig. 1a). TLSs rarely existed near adjacent normal epithelium, and exhibited low-density and small lymphatic clusters around intraepithelial neoplasia. We assessed the TLS density around adjacent normal, dysplastic, and tumoral epithelium in several pT1 cases (Supplementary Fig. S1A). The TLS density (/mm^2^, [range]) in normal, dysplasia, and tumour lesion were 0.058 [0–0.12], 0.10 [0–0.41] and 0.36 [0.091–1.70], respectively. Evaluation of TLS surrounding dysplasia lesions by H&E staining and IHC of CD8, PD-1, CD21 and CD23 were shown in Supplementary Fig. S1B as representative images; matured TLSs were found to existed beneath the dysplasia lesion with the accumulation of TILs expressing CD8 or PD-1 in both TLSs and dysplasia area. Based on these findings, we decided to focus on TLSs in the peritumoral regions for analysis, as described above. In IHC pathology, clusters of CD21^+^ cells were detected inside of PFL-TLSs and SFL-TLSs but not E-TLSs, and CD23^+^ cells further inside, as germinal centres, only of SFL-TLSs (Fig. 1b). We identified peritumoral TLSs in 90.8% of all cases—including E-TLSs in 74.7%, PFL-TLSs in 54.1%, and SFL-TLSs in 64.9%.

Next, we assessed the distribution of peritumoral TLS density and proportion in each maturation category (Fig. 1c). All cases were classified into two groups—TLS high versus TLS low—using the median total TLS density (0.325/mm^2^) as the cut-off value. Compared to the TLS low group, the TLS high group had a significantly lower proportion of immature E-TLSs, and a higher proportion of matured PFL-TLSs and SFL-TLSs (Fig. 1d). We also found that greater progression of pT or pStage was associated with lower peritumoral TLS density (Fig. 1e, f).

### Relationship between TLS expression and clinicopathological parameters

The baseline characteristics of all cases are summarised in Supplementary Table S1. We performed a comparison of clinicopathological characteristics according to TLS density (Table 1). Compared to the TLS high group, the TLS low group was significantly associated with advanced tumour status, including pT2–4 (29.1 vs. 68.3%, *P* < 0.0001), pN1–3 (38.0 vs. 56.3%, *P* = 0.0016), pStage III/IV (49.4 vs. 75.3%, *P* < 0.0001), lymphatic positivity (50.6 vs. 66.5%, *P* = 0.0060) and vascular invasion (34.2 vs. 63.3%, *P* < 0.0001). Additionally, compared to the TLS low group, the TLS high group was associated with significantly better serum nutrition parameters, including serum neutrophil counts (3058 vs. 3674 per mm^3^, *P* < 0.0001), serum albumin (4.0 vs. 3.9, *P* = 0.0059), NLR (1.89 vs. 2.58, *P* < 0.0001), and PNI (48.3 vs. 45.8, *P* = 0.0013), presumably reflecting the systemic immune response. Serum lymphocyte counts tended to be higher in the TLS high group; however, it was not statistically significant (1577 vs. 1452 per mm^3^, *P* = 0.097).

**Table 1:** Clinicopathological characteristics according to density of tertiary lymphoid structures (TLSs).

Characteristics	TLS high group (*n* = 158)	TLS low group (*n* = 158)	*P* value
Age in years, median (range), y	67 (44–90)	69 (43–90)	0.25
Sex			1.00
Male	128 (81.1)	127 (80.4)	
Female	30 (19.0)	31 (19.6)	
Tumour location			0.18
Ut	23 (14.6)	33 (20.9)	
Mt/Lt	135 (85.4)	125 (79.1)	
Histological differentiation (SCC)			0.43
Well/moderate	138 (87.3)	132 (83.5)	
Poor/others	20 (12.7)	26 (16.5)	
pT			<0.0001
T1	112 (70.9)	50 (31.7)	
T2–4	46 (29.1)	108 (68.3)	
pN			0.0016
N0	98 (62.0)	69 (43.7)	
N1–3	60 (38.0)	89 (56.3)	
pM			0.17
M0	154 (97.5)	148 (93.7)	
M1	4 (2.5)	10 (6.3)	
pStage			<0.0001
Stage I/II	80 (50.6)	39 (24.7)	
Stage III/IV	78 (49.4)	119 (75.3)	
Lymphatic invasion			0.0060
Negative	78 (49.4)	53 (33.5)	
Positive	80 (50.6)	105 (66.5)	
Vascular invasion			<0.0001
Negative	104 (65.8)	58 (36.7)	
Positive	54 (34.2)	100 (63.3)	
Lymphocytes in blood, median (range), /mm^3^	1577 (482–3286)	1452 (579–3229)	0.097
Neutrophils in blood, median (range), /mm^3^	3058 (1099–8062)	3674 (1232–10000)	<0.0001
Serum albumin, median (range), g/dL	4.0 (2.6–4.9)	3.9 (2.4–4.7)	0.0059
NLR, median (range)	1.89 (0.58–8.43)	2.58 (0.70–12.74)	<0.0001
PNI, median (range)	48.3 (29.5–59.5)	45.8 (26.9–58.8)	0.013

*Ut* upper thoracic oesophagus, *Mt* middle thoracic oesophagus, *Lt* lower thoracic oesophagus, *SCC* squamous cell carcinoma, *NLR* neutrophil-to-lymphocyte ratio, *PNI* prognostic nutritional index.

Data presented as *n* (%) unless noted otherwise.

### Prognostic impact of TLS expression and maturity in EC patients

**Fig. 2:**
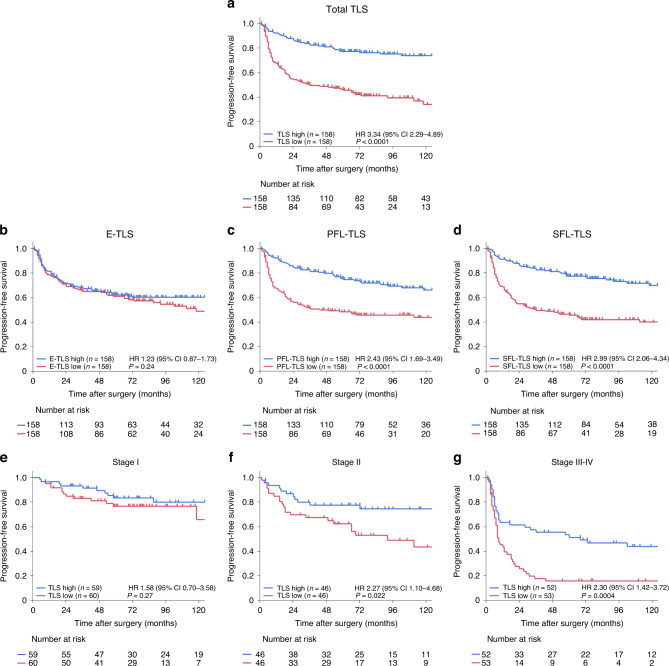
Survival analysis according to tertiary lymphoid structure (TLS) expression in oesophageal cancer patients. **a–d** Kaplan–Meier survival curves for progression-free survival (PFS) in all 316 patients according to the density of total TLSs (**a**), E-TLSs (**b**), PFL-TLSs (**c**), and SFL-TLSs (**d**). **e–g** Subgroup analysis of Kaplan–Meier survival curves for PFS according to total TLS density among Stage I (**e**), Stage II (**f**) and Stage III–IV (**g**) patients. Comparisons were made by the log-rank test. The hazard ratio (HR) and 95% confidence intervals were computed by a Cox proportional hazard model.

Figure 2 presents the survival curves of progression-free survival (PFS) in the TLS high and TLS low groups. Among all TLSs, the TLS high group had a significantly longer PFS compared to the TLS low group (2-year PFS of 81.1% vs. 48.9%, *P* < 0.0001). Regarding TLS maturity, the largest survival difference between the two groups was observed among SFL-TLSs (2-year PFS of 81.1% vs. 48.8%, *P* < 0.0001), followed by PFL-TLSs (2-year PFS of 79.9% vs. 50.0%, *P* < 0.0001), while no difference was observed among E-TLSs (2-year PFS of 65.2% vs. 65.0%, *P* = 0.24) (Fig. 2a–d). In terms of the prognostic impact of TLSs according to pathological stages, compared to the TLS low group, the TLS high group was associated with significantly prolonged PFS in Stage II (HR = 2.27, 95% CI = 1.10–4.68, *P* = 0.022) and Stage III–IV (HR = 2.30, 95% CI = 1.42–3.72, *P* = 0.0004), but not Stage I (HR = 1.58, 95% CI = 0.70–3.58, *P* = 0.27) (Fig. 2e–g). The PFS data classified by TLS density according to pT and pN are shown in Supplementary Fig. S2.

Univariate analysis revealed that PFS was significantly associated with age, location, histological differentiation, pT, pN, pM, lymphatic invasion, vascular invasion and TLS density (Table 2). Multivariate analysis showed that PFS was significantly associated with TLS density (HR = 2.31, 95% CI = 1.55–3.46, *P* < 0.0001), pT (HR = 2.22, 95% CI = 1.46–3.37, *P* = 0.0002), pN (HR = 2.23, 95% CI = 1.52–3.27, *P* < 0.0001), tumour location (HR = 2.09, 95% CI = 1.37–3.21, *P* = 0.0007), and histological differentiation (HR = 1.72, 95%CI = 1.11–2.66, *P* = 0.014) (Table 2). Similar results were obtained in univariate and multivariate analysis for overall survival (Supplementary Fig. S3 and Supplementary Table S2).

**Table 2:** Univariate and multivariate analysis of progression-free survival.

	Univariate analysis		Multivariate analysis	
	HR (95% CI)	*P* value	HR (95% CI)	*P* value
Age		0.0007		0.011
<68 years	1 [Reference]		1 [Reference]	
≥68 years	1.86 (1.30–2.66)		1.60 (1.12–2.29)	
Sex		0.27	NA	NA
Male	1.30 (0.82–2.08)			
Female	1 [Reference]			
Location		0.024		0.0007
Ut	1.60 (1.06–2.42)		2.09 (1.37–3.21)	
Mt/Lt	1 [Reference]		1 [Reference]	
Histological differentiation (SCC)		0.014		0.014
Well/moderate	1 [Reference]		1 [Reference]	
Poor/others	1.72 (1.12–2.64)		1.72 (1.11–2.66)	
pT		<0.0001		0.0002
T1	1 [Reference]		1 [Reference]	
T2–4	3.53 (2.41–5.17)		2.22 (1.46–3.37)	
pN		<0.0001		<0.0001
N0	1 [Reference]		1 [Reference]	
N1–3	2.81 (1.95–4.04)		2.23 (1.52–3.27)	
pM		0.0026		0.10
M0	1 [Reference]		1 [Reference]	
M1	2.71 (1.42–5.17)		1.74 (0.89–3.37)	
TLS density		<0.0001		<0.0001
High	1		1	
Low	3.35 (2.29–4.90)		2.31 (1.55–3.46)	

*HR* hazard ratio, *CI* confidence interval, *NA* not applicable, *Ut* upper thoracic oesophagus, *Mt* middle thoracic oesophagus, *Lt* lower thoracic oesophagus, *SCC* squamous cell carcinoma.

### Cellular composition of peritumoral TLSs per their maturity as assessed by multiplex IF

**Fig. 3:**
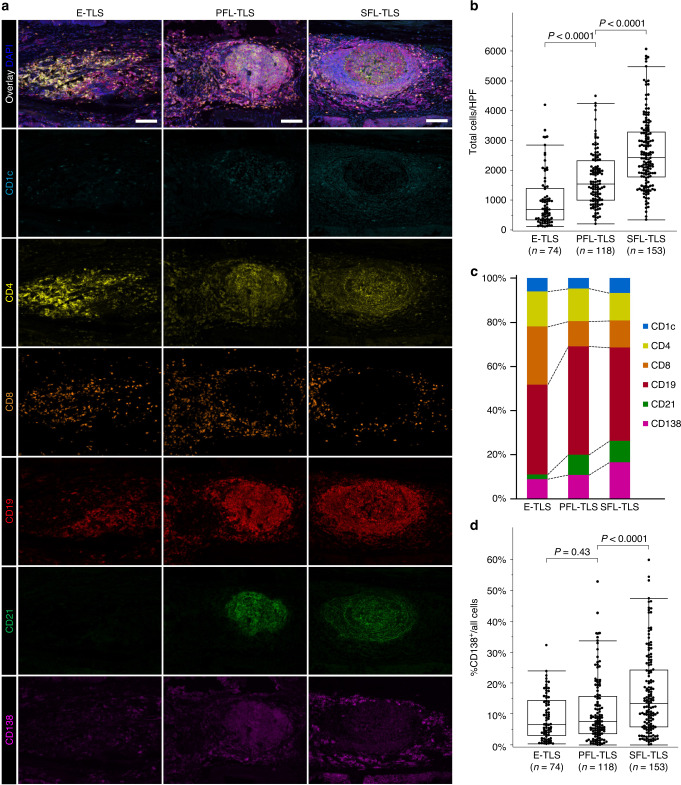
Evaluation of tertiary lymphoid structures (TLSs) using multiplex immunofluorescence in 70 oesophageal cancer patients. **a** Representative images of each TLS maturation category, showing DCs (CD1c, light blue), CD4 T cells (CD4, yellow), CD8 T cells (CD8, orange), B cells (CD19, red), follicular DCs (CD21, green), plasma cells (CD138, purple) and DAPI. **b** Total cell counts in high-power field (HPF) based on TLS maturation category. **c** Composition of six immune cell subsets in TLSs according to each maturation category. **d** Proportion of CD138^+^ plasma cells in TLSs according to each maturation category. Scale bars = 200 µm. Comparisons were made by Mann–Whitney *U* test.

We used multiplex IF to further assess cellular composition in a total of 345 peritumoral TLSs (74 E-TLSs, 118 PFL-TLSs, and 153 SFL-TLSs) from 70 cases, according to their maturation categories. Figure 3a shows representative pictures of multiplex IF for each antibody, and the merged images, according to TLS maturation categories. With increasing maturity of peritumoral TLSs, the total positive cell count per high-power field (HPF) gradually increased. The median count (range) was 681.5 (115–4197) in E-TLSs, 1546.5 (207–4496) in PFL-TLSs, and 2427 (350–6072) in SFL-TLSs (Fig. 3b). In addition, the proportion of component cell types differed among the different maturation categories (Fig. 3c). With increasing maturity of peritumoral TLSs, the proportion of CD8^+^ T lymphocytes decreased, while the proportion of CD21^+^ follicular dendritic cells obviously increased. Notably, the proportion of CD138^+^ plasma cells was significantly associated with TLS maturity (6.6% in E-TLSs, 7.5% in PFL-TLSs, and 13.4% in SFL-TLSs; *P* < 0.0001 in PFL-TLSs vs. SFL-TLSs) (Fig. 3d). Supplementary Fig. S4 shows the detailed distributions of other cells.

### Predictive value of TLS status for treatment response and prognosis of anti-PD-1 antibody therapy in EC patients

**Fig. 4:**
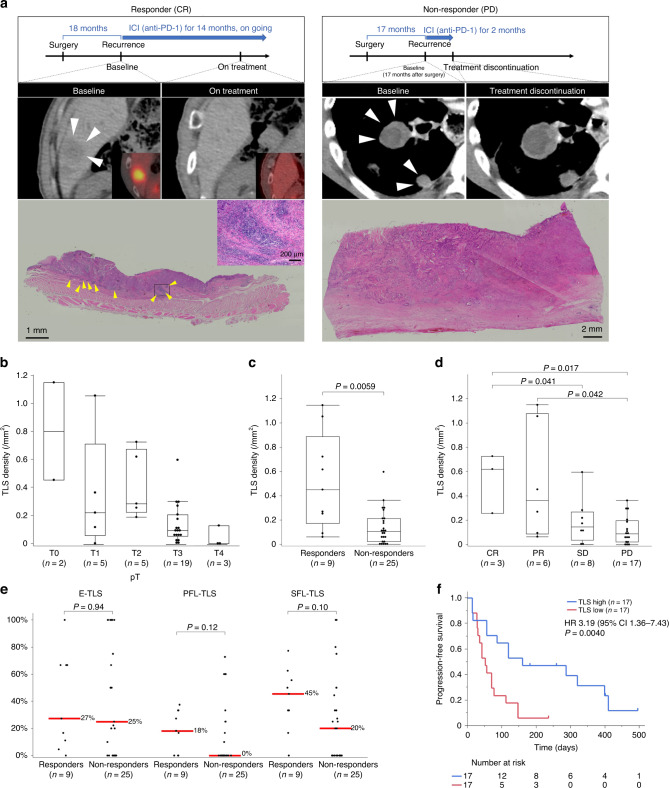
Evaluation of tertiary lymphoid structure (TLS) density and maturity in resected specimens from prior surgery, in association with response to anti-PD-1 antibody and survival in a different cohort of 34 patients with recurrent oesophageal cancer. **a** Scheme of treatment course and CT/PET-CT images of target lesions (white arrowheads), and H&E images of TLS (yellow arrowheads) in whole tumour sections from a representative responder (left) and non-responder (right) to anti-PD-1 antibody. **b** TLS density according to pT in recurrent oesophageal cancer patients treated with anti-PD-1 antibody. **c**, **d** TLS density according to two (**c**) and four (**d**) categories of response to anti-PD-1 antibody. **e** Comparison of TLS maturity according to response to anti-PD-1 antibody. Red lines and the numbers next to them show median. **f** Kaplan–Meier survival curve of PFS according to TLS density in resected specimens from prior surgery. Comparisons were made by Mann–Whitney *U* test for continuous variables, and a log-rank test for survival analysis.

We further examined the predictive values of peritumoral TLS in a different cohort of recurrent EC patients treated with anti-PD-1 antibody monotherapy. The baseline characteristics of these patients according to TLS density and treatment response to anti-PD-1 antibodies are summarised in Supplementary Tables S3 and S4, respectively. Figure 4a shows histological images of peritumoral TLSs in resected specimens of representative cases (responders versus non-responders). Supplementary Fig. S5 illustrates the overall distribution of TLS density and maturity. TLS density tended to decrease along with the tumour progression in ICI-treated cases (Fig. 4b). Remarkably, TLS density was significantly correlated with clinical response to anti-PD-1 antibody therapy, in terms of responders vs. non-responders (0.45 vs. 0.10 per mm^2^, *P* = 0.0070), and the four categories of complete response (0.62/mm^2^), partial response (0.36/mm^2^), stable disease (0.089/mm^2^), and progressive disease (0.021/mm^2^) (Fig. 4c, d). The comparison of TLS maturity in the two group was shown in Fig. 4e. Survival analysis in this cohort revealed that compared to the TLS low group, the TLS high group was associated with significantly better median PFS (160 vs. 52 days, *P* = 0.0040) (Fig. 4f). Evaluation of PD-1+ TILs in the resected specimen of 34 patients with recurrent EC is shown as a representative image in Supplementary Fig. S6; the high expression of PD-1+ TILs were observed within or around tumour with high density of TLSs. The PFS data according to PD-L1 expression (tumour proportion score; TPS and combined positive score; CPS) were shown in Supplementary Fig. S7. We found that the higher expression of PD-L1 (i.e., higher TPS and CPS) was associated with the favourable PFS although it did not significantly correlate with response to ICI treatment (Supplementary Table S4).

## Discussion

In this study, we quantitatively and objectively assessed peritumoral TLS expression and maturity in preoperatively untreated resected specimens from a large series of over 300 EC cases from two institutes. TLS density, which is positively correlated with TLS maturity, was found to be associated with tumour stage, and with serum nutrition markers, presumably reflecting the systemic immune response. Moreover, TLS maturity progression was associated with an increasing number of constitutive cells, particularly a remarkable increase of CD138^+^ plasma cells. TLS density was identified as an independent prognostic factor in the primary EC cohort. Remarkably, in a different cohort of patients with recurrent EC, TLS density in resected specimens from prior surgery predicted the clinical response to anti-PD-1 antibody and patient survival. To our knowledge, this is the first to demonstrate clinical significance of peritumoral TLS in predicting the prognosis and the efficacy of anti-PD-1 antibody treatment in a large cohort of EC patients.

The present results elucidated the heterogeneity of TLS expression, localisation, and maturation in EC. Previous studies have reported that high expression of TLSs is associated with tumour stage or favourable prognosis in other cancer types, including gastric and colorectal cancer [31, 32]. On the other hand, Ding et al. observed that high peritumoral TLS expression was correlated with unfavourable patient survival in intrahepatic cholangiocarcinoma [33]. Regarding maturity, Posch et al. found that TLS maturation had prognostic value and was a predictive factor for colorectal cancer recurrence [34]. Deguchi et al. showed that EC with abundant matured TLSs defined as the presence of germinal centre (GC-TLSs) showed a better prognosis compared with those with fewer matured TLSs in cStage III–IV cases treated with neoadjuvant chemotherapy [35]. In this report, they also found the association between GC-TLSs and treatment response to neoadjuvant chemotherapy although the clinical impact of TLSs remains unclear in EC patients with ICI treatment. In lung cancer, Silina et al. showed that matured TLSs were associated with a favourable prognosis, and that the number of matured TLSs decreased after chemotherapy and radiotherapy [21]. TLS expression, location, and maturation varies—and may have different influences on anti-tumour immunity and patient prognosis—depending on involved organs or cancer types. This study showed that the expression and maturation of peritumoral TLSs contributed to the anti-tumour effect in EC patients, as shown in other cancer types.

The presently observed inverse correlation between peritumoral TLS density and tumour progression may suggest several possible mechanisms. TLS formation might inhibit tumour growth, which is supported by previous reports demonstrating improved prognosis in cases with abundant tumour antigen-specific or active cytotoxic lymphocytes associated with high TLS expression [36–38]. The accumulations of TILs along with dysplasia-associated TLSs may indicate that TLSs, formed during the process of carcinogenesis, activate antitumor immunity as represented by the proliferation and infiltration of antigen-specific lymphocytes, implying that TLSs play an important role of immunosurveillance against EC carcinogenesis. Another possibility is that tumours suppress TLS formation, based on previous evidence that TME formation is suppressed by tumour-derived products, i.e., cancer antigens, driver gene mutations, etc. [39]. However, these proposed mechanisms are controversial and must be clarified in future studies. Intriguingly, the present study revealed a significant correlation between peritumoral TLS density and host factors, including age and serum nutritional/immunological indices, which supports previous reports in gastric cancer [40]. These findings may imply that relatively young patients with potentially better nutritional status may have greater antitumor immune response activation, inducing a larger number of tumour-associated TLSs [41, 42]. In addition, our findings might support the use of nutritional and rehabilitative interventions in EC patients who are likely to suffer from serious weight loss and cachexia due to tumoral obstruction.

In this study, the TLS high group contained abundant matured TLSs. Previous reports described that the B cells in matured TLSs promote tumour-specific antibody production and T-cell activation, whereas immature TLSs may inhibit immune responses by producing suppressive molecules [43]. Our present IF results showed a markedly increased CD138^+^ plasma cells in matured TLSs. Plasma cells are specialised for producing antigen-specific or high-affinity antibodies, and thus play an important role in humoral immunity. The correlation between tumour-infiltrating plasma cells and favourable prognosis has been demonstrated in several cancer types, including EC [36, 44]. Previous researches also provide evidence of antibody-dependent tumour cell death mediated by TLSs and surrounding plasma cells, and its association with favourable survival among cancer patients treated with ICI [45, 46]. Our results could support these previous findings, although we did not evaluate the antibody productivity or antigen specificity of plasma cells. Further studies are needed to clarify the mechanism of B-cell differentiation in TLSs.

Our present analyses of the recurrent EC cohort revealed that a high density of peritumoral TLSs in primary tumours predicted the response to later treatment with anti-PD-1 antibody, and subsequent survival, indicating clinical benefit of TLS evaluation in the initial specimen. Previous studies in other cancer types have also shown that baseline TLS expression in a pretreatment biopsy is associated with the outcome of ICI treatment [18–20, 47]. A possible mechanism explaining these findings could be that abundant peritumoral TLSs are associated with increased tumour-associated memory B cells, plasma cells, or PD-1^+^ immune infiltrates [48, 49]. The baseline priming of immune cells in TLSs may promote greater diversity and capacity of tumour-specific lymphocytes, resulting in enhancing the response to ICI therapy. Several studies have reported that neoadjuvant chemotherapy or vaccine therapy promotes TLS formation, supporting the concept of combination immunotherapy for cancer treatment, including the combination of ICI with chemotherapy/chemoradiation, which has been clinically used to treat EC patients [17]. Furthermore, there have been several preclinical reports of TLS-inducing interventions in a mouse model [50], which will likely lead to the future establishment of personalised cancer immunotherapy strategies via “TLS induction”.

This study has several limitations, including the retrospective design, the lack of evaluation of how treatments influenced TLS density/maturity, and the fact that our results were based on evaluation of only single slide per antibody per case. Moreover, since our histological evaluation was confined to the TLSs themselves or the adjacent areas, we did not assess any interactions between TLSs and other infiltrates throughout the tumours. Further studies are needed to assess the effecter cells, such as for the activation/exhaustion profiles, cytokine secretion capacity, or receptor repertoire.

To conclude, peritumoral TLS density in resected specimens from EC patients was significantly correlated with serum nutrition parameters, and was associated with tumour stage and identified as an independent prognostic factor. Notably, matured TLS exhibited high proportions of CD138^+^ plasma cells, and showed particularly significant clinical relevance. Since TLSs appear to predict prognosis and clinical efficacy of anti-PD-1 antibody therapy, they could be a useful biomarker for personalised multimodal treatments, including immunotherapy, for EC patients.

## Supplementary information

Supplementary Table Legends

Supplementary Table S1

Supplementary Table S2

Supplementary Table S3

Supplementary Table S4

Supplementary Figure Lagends

Supplenmentary Figure S1

Supplementary Figure S2

Supplementary Figure S3

Supplementary Figure S4

Supplementary Figure S5

Supplementary Figure S6

Supplementary Figure S7

## Data Availability

All data were generated by the authors and are available upon request to the corresponding authors of this study.
