# Supplementary material for: Density and maturity of peritumoral tertiary lymphoid structures in oesophageal squamous cell carcinoma predicts patient survival and response to immune checkpoint inhibitors
Source: Br J Cancer. 2023 Apr 4;128(12):2175–85. doi: 10.1038/s41416-023-02235-9 (PMC10241865; doi:10.1038/s41416-023-02235-9)
Supplement: Supplementary file 1 — Supplementary Table Legends [file 41416_2023_2235_MOESM1_ESM.docx]

# Supplementary Table Legends

**Supplementary Table S1**

Baseline clinicopathological characteristics of all patients with oesophageal cancer.

Data are presented as n (%) unless otherwise noted. Abbreviations: Ut, upper thoracic esophagus; Mt, middle thoracic esophagus; Lt, lower thoracic esophagus; SCC, squamous cell carcinoma; NLR, neutrophil to lymphocyte ratio; PNI, prognostic nutritional index.

**Supplementary Table S2**

Univariate and multivariate analysis of overall survival.

Abbreviations: HR, hazard ratio; CI, confidence interval; NA, not applicable; Ut, upper thoracic esophagus; Mt, middle thoracic esophagus; Lt, lower thoracic esophagus; SCC, squamous cell carcinoma; TLS, tertiary lymphoid structure.

**Supplementary Table S3.**

Baseline clinicopathological characteristics according to density of tertiary lymphoid structures (TLSs) in patients with anti-PD-1 antibody treatment for recurrent tumor.

Data presented as n (%) unless noted otherwise. Abbreviations: Ut, upper thoracic esophagus; Mt, middle thoracic esophagus; Lt, lower thoracic esophagus; SCC, squamous cell carcinoma; CR, complete response; PR, partial response; SD stable disease; PD progression disease; TPS, Tumor proportion score; CPS, combined positive score.

**Supplementary Table S4.**

Baseline clinicopathological characteristics according to treatment response to anti-PD-1 antibody treatment for recurrent tumor.

Data presented as n (%) unless noted otherwise. Abbreviations: Ut, upper thoracic esophagus; Mt, middle thoracic esophagus; Lt, lower thoracic esophagus; SCC, squamous cell carcinoma; CR, complete response; PR, partial response; SD stable disease; PD progression disease; TPS, Tumor proportion score; CPS, combined positive score.
