# Supplementary material for: Density and maturity of peritumoral tertiary lymphoid structures in oesophageal squamous cell carcinoma predicts patient survival and response to immune checkpoint inhibitors
Source: Br J Cancer. 2023 Apr 4;128(12):2175–85. doi: 10.1038/s41416-023-02235-9 (PMC10241865; doi:10.1038/s41416-023-02235-9)
Supplement: Supplementary file 2 — Supplementary Table S1 [file 41416_2023_2235_MOESM2_ESM.docx]

**Supplementary Table S1. Baseline clinicopathological characteristics of all patients with oesophageal cancer.**

| Characteristics | No. (%)  (n = 316) |
| --- | --- |
| Age in years, median (range), y | 68 (43–90) |
| Sex  Male  Female | 255 (80.7)  61 (19.3) |
| Tumor Location  Ut  Mt  Lt | 56 (17.7)  169 (53.5)  91 (28.8) |
| Histological differentiation (SCC)  well  moderate  poor  others | 62 (19.6)  208 (65.8)  40 (12.7)  6 (1.9) |
| pT  T1  T2  T3  T4 | 162 (51.3)  40 (12.7)  103 (32.6)  11 (3.5) |
| pN  N0  N1  N2  N3 | 167 (52.8)  86 (27.2)  44 (13.9)  19 (6.0) |
| pM  M0  M1 | 302 (95.6)  14 (4.4) |
| pStage  Stage I  Stage II  Stage III  Stage IV | 119 (37.7)  92 (29.1)  77 (24.4)  28 (8.9) |
| Lymphatic invasion  negative  positive | 131 (41.5)  185 (58.5) |
| Vascular invasion  negative  positive | 162 (51.3)  154 (48.7) |
| Lymphocytes in blood, median (range), /mm^3^ | 1503 (482–3286) |
| Neutrophils in blood, median (range), /mm^3^ | 3369 (1099–10000) |
| Serum albumin, median (range), g/dL | 3.9 (2.4–4.9) |
| NLR, median (range) | 2.22 (0.58–12.74) |
| PNI, median (range) | 47.1 (26.9–59.5) |

Data are presented as n (%) unless otherwise noted.

Abbreviations: Ut, upper thoracic esophagus; Mt, middle thoracic esophagus; Lt, lower thoracic esophagus; SCC, squamous cell carcinoma; NLR, neutrophil to lymphocyte ratio; PNI, prognostic nutritional index.
