# Supplementary material for: Density and maturity of peritumoral tertiary lymphoid structures in oesophageal squamous cell carcinoma predicts patient survival and response to immune checkpoint inhibitors
Source: Br J Cancer. 2023 Apr 4;128(12):2175–85. doi: 10.1038/s41416-023-02235-9 (PMC10241865; doi:10.1038/s41416-023-02235-9)
Supplement: Supplementary file 3 — Supplementary Table S2 [file 41416_2023_2235_MOESM3_ESM.docx]

**Supplementary Table S2. Univariate and multivariate analysis of overall survival.**

|  | Univariate analysis | |  | Multivariate analysis | |
| --- | --- | --- | --- | --- | --- |
|  | HR (95% CI) | *P* value |  | HR (95% CI) | *P* value |
| Age  <68 years  ≥68 years | 1 [Reference]  1.68 (1.13–2.50) | .010 |  | 1 [Reference]  1.40 (0.93–2.08) | .10 |
| Sex  Male  Female | 1.23 (0.73–2.07)  1 [Reference] | .44 |  | NA | NA |
| Location  Ut  Mt/Lt | 1.56 (0.99–2.48)  1 [Reference] | .057 |  | NA | NA |
| Histological differentiation (SCC)  well/moderate  poor/others | 1 [Reference]  2.11 (1.33–3.33) | .014 |  | 1 [Reference]  2.06 (1.30–3.27) | .0022 |
| pT  T1  T2–4 | 1 [Reference]  3.57 (2.30–5.55) | <.0001 |  | 1 [Reference]  1.75 (1.30–3.40) | .0026 |
| pN  N0  N1–3 | 1 [Reference]  2.55 (1.70–3.83) | <.0001 |  | 1 [Reference]  1.75 (1.14–2.68) | .0098 |
| pM  M0  M1 | 1 [Reference]  3.00 (1.51–5.97) | .0017 |  | 1 [Reference]  2.22 (1.10–4.49) | .026 |
| TLS density  high  low | 1 [Reference]  3.52 (2.27–5.45) | <.0001 |  | 1 [Reference]  2.41 (1.51–3.84) | .0002 |

Abbreviations: HR, hazard ratio; CI, confidence interval; NA, not applicable; Ut, upper thoracic esophagus; Mt, middle thoracic esophagus; Lt, lower thoracic esophagus; SCC, squamous cell carcinoma; TLS, tertiary lymphoid structure.
