# Supplementary material for: Density and maturity of peritumoral tertiary lymphoid structures in oesophageal squamous cell carcinoma predicts patient survival and response to immune checkpoint inhibitors
Source: Br J Cancer. 2023 Apr 4;128(12):2175–85. doi: 10.1038/s41416-023-02235-9 (PMC10241865; doi:10.1038/s41416-023-02235-9)
Supplement: Supplementary file 4 — Supplementary Table S3 [file 41416_2023_2235_MOESM4_ESM.docx]

**Supplementary Table S3. Baseline clinicopathological characteristics according to density of tertiary lymphoid structures (TLSs) in patients with anti-PD-1 antibody treatment for recurrent tumor.**

| Characteristics | TLS high group  (n = 17) | TLS low group  (n = 17) | *P* value |
| --- | --- | --- | --- |
| Age in years, median (range), y | 70 (52–83) | 72 (49–83) | .72 |
| Sex  Male  Female | 12 (70.6)  5 (29.4) | 15 (88.2)  2 (11.8) | .40 |
| Tumor Location  Ut  Mt/Lt | 4 (23.5)  13 (76.5) | 3 (17.7)  14 (82.3) | 1.00 |
| Histological differentiation (SCC)  well  moderate | 1 (5.9)  16 (94.1) | 0  17 (100) | 1.00 |
| cT of primary tumor  T1—2  T3–4 | 5 (29.4)  12 (70.6) | 1 (5.9)  16 (94.1) | .17 |
| cN of primary tumor  N0  N1–3 | 3 (17.7)  14 (82.4) | 1 (5.9)  16 (94.1) | .60 |
| cM of primary tumor  M0  M1 | 12 (70.6)  5 (29.4) | 17 (100)  0 | .17 |
| cStage of primary tumor  Stage I/II  Stage III/IV | 3 (17.7)  14 (82.4) | 1 (5.9)  16 (94.1) | .60 |
| pT of primary tumor  T1—2  T3–4 | 10 (58.8)  7 (41.2) | 2 (11.8)  15 (88.2) | .010 |
| pN of primary tumor  N0  N1–3 | 5 (29.4)  12 (70.6) | 2 (11.8)  15 (88.2) | .40 |
| pM of primary tumor  M0  M1 | 13 (76.5)  4 (23.5) | 17 (100)  0 | .10 |
| pStage  Stage I/II  Stage III/IV | 4 (23.5)  13 (76.5) | 3 (17.7)  14 (82.4) | 1.00 |
| Lymphatic invasion  negative  positive | 8 (47.1)  9 (52.9) | 4 (23.5)  13 (76.5) | .28 |
| Vascular invasion  negative  positive | 12 (70.6)  5 (29.4) | 7 (41.2)  10 (58.8) | <.0001 |
| Clinical response to anti-PD-1 antibody  CR/PR  SD/PD | 8 (47.1)  9 (52.9) | 1 (5.9)  16 (94.1) | 0.017 |
| PD-L1 expression (TPS)  ≥ 1%  < 1% | 12 (70.6)  5 (29.4) | 6 (35.3)  11 (64.7) | .084 |
| PD-L1 expression (TPS)  ≥ 10%  < 10% | 9 (52.9)  8 (47.1) | 3 (17.7)  14 (82.4) | .071 |
| PD-L1 expression (CPS)  ≥ 1%  < 1% | 17 (100)  0 | 14 (82.3)  3 (17.7) | .23 |
| PD-L1 expression (CPS)  ≥ 5%  < 5% | 12 (70.6)  5 (29.4) | 6 (35.3)  11 (64.7) | .084 |
| PD-L1 expression (CPS)  ≥ 10%  < 10% | 8 (47.1)  9 (52.9) | 2 (11.8)  15 (88.2) | .057 |

Data presented as n (%) unless noted otherwise.
Abbreviations: Ut, upper thoracic esophagus; Mt, middle thoracic esophagus; Lt, lower thoracic esophagus; SCC, squamous cell carcinoma; CR, complete response; PR, partial response; SD stable disease; PD progression disease; TPS, Tumor proportion score; CPS, combined positive score.
