# Supplementary material for: Density and maturity of peritumoral tertiary lymphoid structures in oesophageal squamous cell carcinoma predicts patient survival and response to immune checkpoint inhibitors
Source: Br J Cancer. 2023 Apr 4;128(12):2175–85. doi: 10.1038/s41416-023-02235-9 (PMC10241865; doi:10.1038/s41416-023-02235-9)
Supplement: Supplementary file 5 — Supplementary Table S4 [file 41416_2023_2235_MOESM5_ESM.docx]

**Supplementary Table S4. Baseline clinicopathological characteristics according to treatment response to anti-PD-1 antibody treatment for recurrent tumor.**

| Characteristics | Responders  (n = 9) | Non-responders  (n = 25) | *P* value |
| --- | --- | --- | --- |
| Age in years, median (range), y | 70 (60–79) | 71 (49–83) | .58 |
| Sex  Male  Female | 7 (77.8)  2 (22.2) | 20 (80.0)  5 (20.0) | 1.00 |
| Tumor Location  Ut  Mt/Lt | 4 (44.4)  5 (55.6) | 3 (12.0)  22 (88.0) | .061 |
| Histological differentiation (SCC)  well  moderate | 1 (11.1)  8 (88.9) | 0  25 (100) | .26 |
| cT of primary tumor  T1—2  T3–4 | 3 (33.3)  6 (66.7) | 3 (12.0)  22 (88.0) | .31 |
| cN of primary tumor  N0  N1–3 | 1 (11.1)  8 (88.9) | 3 (12.0)  22 (88.0) | 1.00 |
| cM of primary tumor  M0  M1 | 8 (88.9)  1 (11.1) | 20 (80.0)  5 (20.0) | 1.00 |
| cStage of primary tumor  Stage I/II  Stage III/IV | 1 (11.1)  8 (88.9) | 3 (12.0)  22 (88.0) | 1.00 |
| pT of primary tumor  T1—2  T3–4 | 6 (66.7)  3 (33.3) | 6 (24.0)  19 (76.0) | .040 |
| pN of primary tumor  N0  N1–3 | 3 (33.3)  6 (66.7) | 4 (16.0)  21 (884.0) | .35 |
| pM of primary tumor  M0  M1 | 8 (88.9)  1 (11.1) | 22 (88.0)  3 (12.0) | 1.00 |
| pStage  Stage I/II  Stage III/IV | 2 (22.2)  7 (77.8) | 5 (20.0)  20 (80.0) | 1.00 |
| Lymphatic invasion  negative  positive | 7 (77.8)  2 (22.2) | 5 (20.0)  20 (80.0) | .0037 |
| Vascular invasion  negative  positive | 7 (77.8)  2 (22.2) | 12 (48.0)  13 (52.0) | .24 |
| TLS density, /mm^2^, median (range) | 0.45 (0.062–1.15) | 0.10 (0–0.60) | 0.0059 |
| PD-L1 expression (TPS)  ≥ 1%  < 1% | 7 (77.8)  2 (22.2) | 11 (44.0)  14 (56.0) | .13 |
| PD-L1 expression (TPS)  ≥ 10%  < 10% | 4 (44.4)  5 (55.6) | 8 (32.0)  17 (68.0) | .69 |
| PD-L1 expression (CPS)  ≥ 1%  < 1% | 9 (100)  0 | 22 (88.0)  3 (12.0) | .55 |
| PD-L1 expression (CPS)  ≥ 5%  < 5% | 7 (77.8)  2 (22.2) | 11 (44.0)  14 (56.0) | .13 |
| PD-L1 expression (CPS)  ≥ 10%  < 10% | 3 (33.3)  6 (66.7) | 7 (28.0)  18 (72.0) | 1.00 |

Data presented as n (%) unless noted otherwise.
Abbreviations: Ut, upper thoracic esophagus; Mt, middle thoracic esophagus; Lt, lower thoracic esophagus; SCC, squamous cell carcinoma; CR, complete response; PR, partial response; SD stable disease; PD progression disease; TPS, Tumor proportion score; CPS, combined positive score.
