# Supplementary material for: Density and maturity of peritumoral tertiary lymphoid structures in oesophageal squamous cell carcinoma predicts patient survival and response to immune checkpoint inhibitors
Source: Br J Cancer. 2023 Apr 4;128(12):2175–85. doi: 10.1038/s41416-023-02235-9 (PMC10241865; doi:10.1038/s41416-023-02235-9)
Supplement: Supplementary file 6 — Supplementary Figure Lagends [file 41416_2023_2235_MOESM6_ESM.docx]

# Supplementary Figure Legends

**Supplementary Figure S1**

Assessment of tertiary lymphoid structures (TLSs) surrounding normal, dysplasia and early tumor tissues in oesophagus. A, TLS density in normal, dysplasia and pT1 tumor lesions. B, Representative images of TLS in dysplasia lesions, showing hematoxylin/eosin staining and immunohistochemistry of CD8, PD-1, CD21, and CD23. Scale bar = 100 µm. Comparisons were made by Mann-Whitney U test.

**Supplementary Figure S2**

Subgroup analysis of Kaplan-Meier survival curves for PFS according to total TLS density among pathological T1 (**A**), T2 (**B**), T3-T4 (**C**) patients and pathological N0 (**D**), N1-3 (**E**) patients . Comparisons were made by the log-rank test. The hazard ratio (HR) and 95% confidence intervals were computed by a Cox proportional hazard model.

**Supplementary Figure S3.**

Survival analysis according to tertiary lymphoid structure (TLS) expression in oesophageal cancer patients. **A–D**, Kaplan-Meier survival curves for overall survival (OS) in all 316 patients, according to density of total TLSs (**A**), E-TLSs (**B**), PFL-TLSs (**C**), and SFL-TLSs (**D**). **E–G**, Subgroup analysis of Kaplan-Meier survival curve for PFS according to total TLS density in Stage I (**E**), Stage II (**F**), and Stage III–IV (**G**) patients. Comparisons were made by the log-rank test. The hazard ratio (HR) and 95% confidence intervals were computed by a Cox proportional hazard model.

**Supplementary Figure S4.**

Evaluation of tertiary lymphoid structures (TLSs) using multiplex immunofluorescence. Proportions of other component immune cells, except plasma cells, in TLSs according to each maturation category. **A**, DCs. **B**, CD4 T cells. **C**, CD8 T cells. **D**, B cells. **E**, follicular DCs.

**Supplementary Figure S5.**

The distribution of tertiary lymphoid structure (TLS) density and the proportion of each TLS maturation category among 34 patients with recurrent oesophageal cancer treated with anti-PD-1 antibody monotherapy.

**Supplementary Figure S6**

Representative images of tumor infiltrating lymphocytes in serial tumor sections of a patient treated with anti-PD-1 antibody treatment. **A**, Hematoxylin/eosin staining. **B**, PD-1 immunohistochemistry. Scale bar = 100 µm.

**Supplementary Figure S7**

Survival analysis according to PD-L1 expression in patients with recurrent oesophageal cancer treated with anti-PD-1 antibody therapy. **A–F,** Kaplan-Meier survival curve of progression-free survival according to each cut off of CPS = 1% (**A**), CPS = 5% (**B**), CPS = 10% (**C**), TPS = 1% (**D**), TPS = 5% (**E**), and TPS = 10% (**F**). Comparisons were made by a log-rank test. The hazard ratio (HR) and 95% confidence intervals were computed by a Cox proportional hazard model.
