# Supplementary figures and images for: Density and maturity of peritumoral tertiary lymphoid structures in oesophageal squamous cell carcinoma predicts patient survival and response to immune checkpoint inhibitors
Source: Br J Cancer. 2023 Apr 4;128(12):2175–85. doi: 10.1038/s41416-023-02235-9 (PMC10241865; doi:10.1038/s41416-023-02235-9)

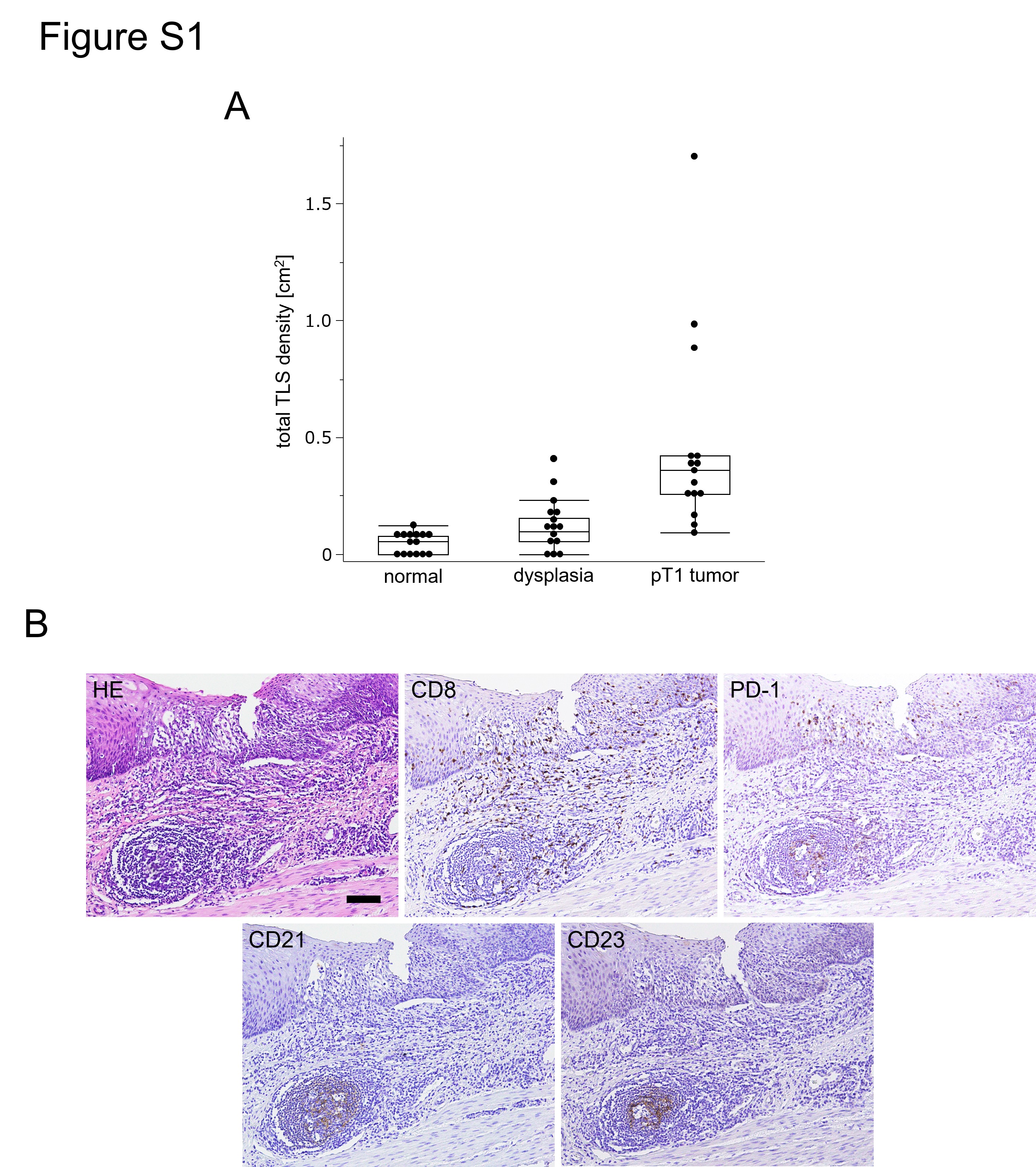

Supplement: Supplementary file 7 — Supplenmentary Figure S1 [file 41416_2023_2235_MOESM7_ESM.jpg]

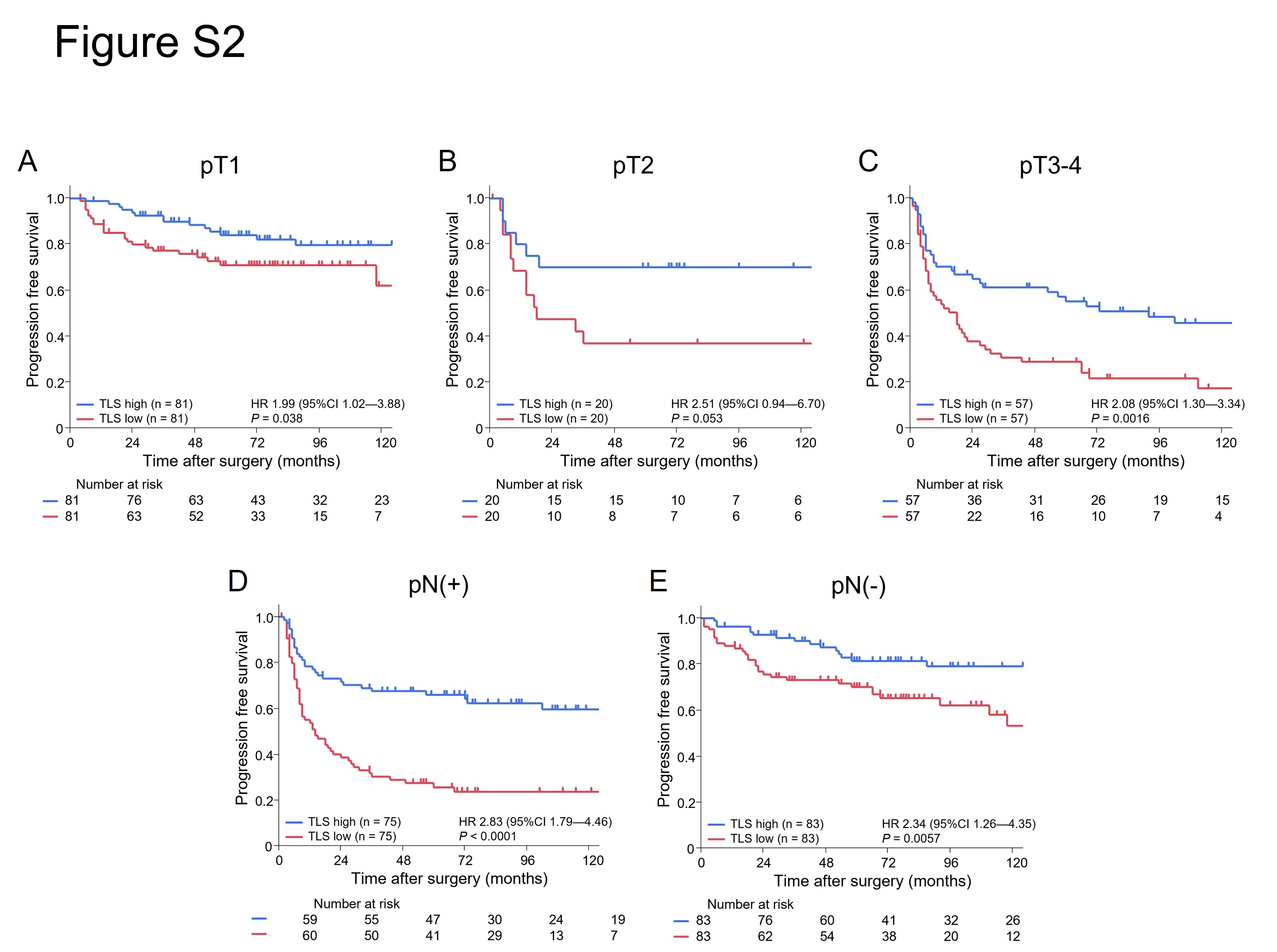

Supplement: Supplementary file 8 — Supplementary Figure S2 [file 41416_2023_2235_MOESM8_ESM.jpg]

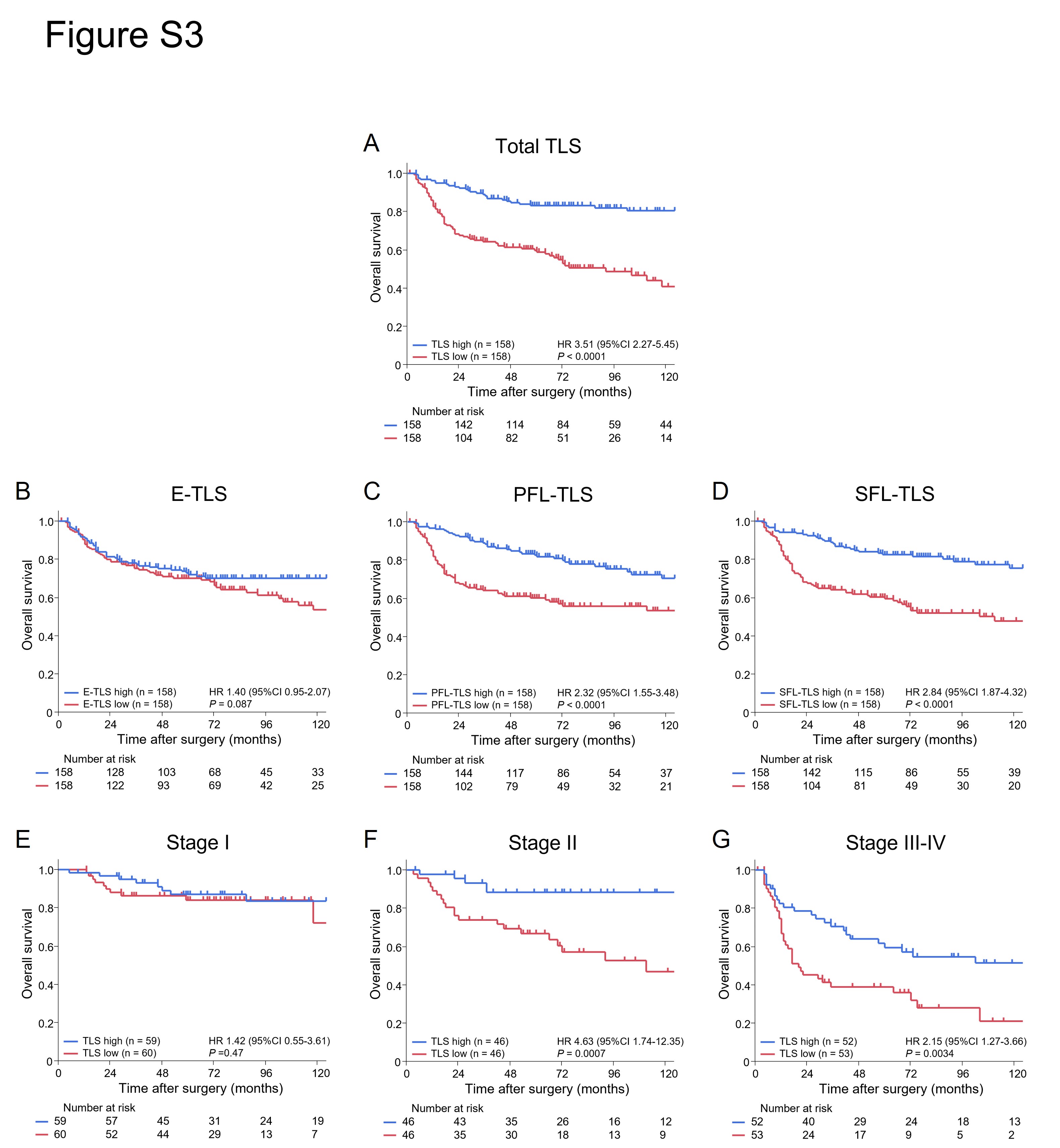

Supplement: Supplementary file 9 — Supplementary Figure S3 [file 41416_2023_2235_MOESM9_ESM.jpg]

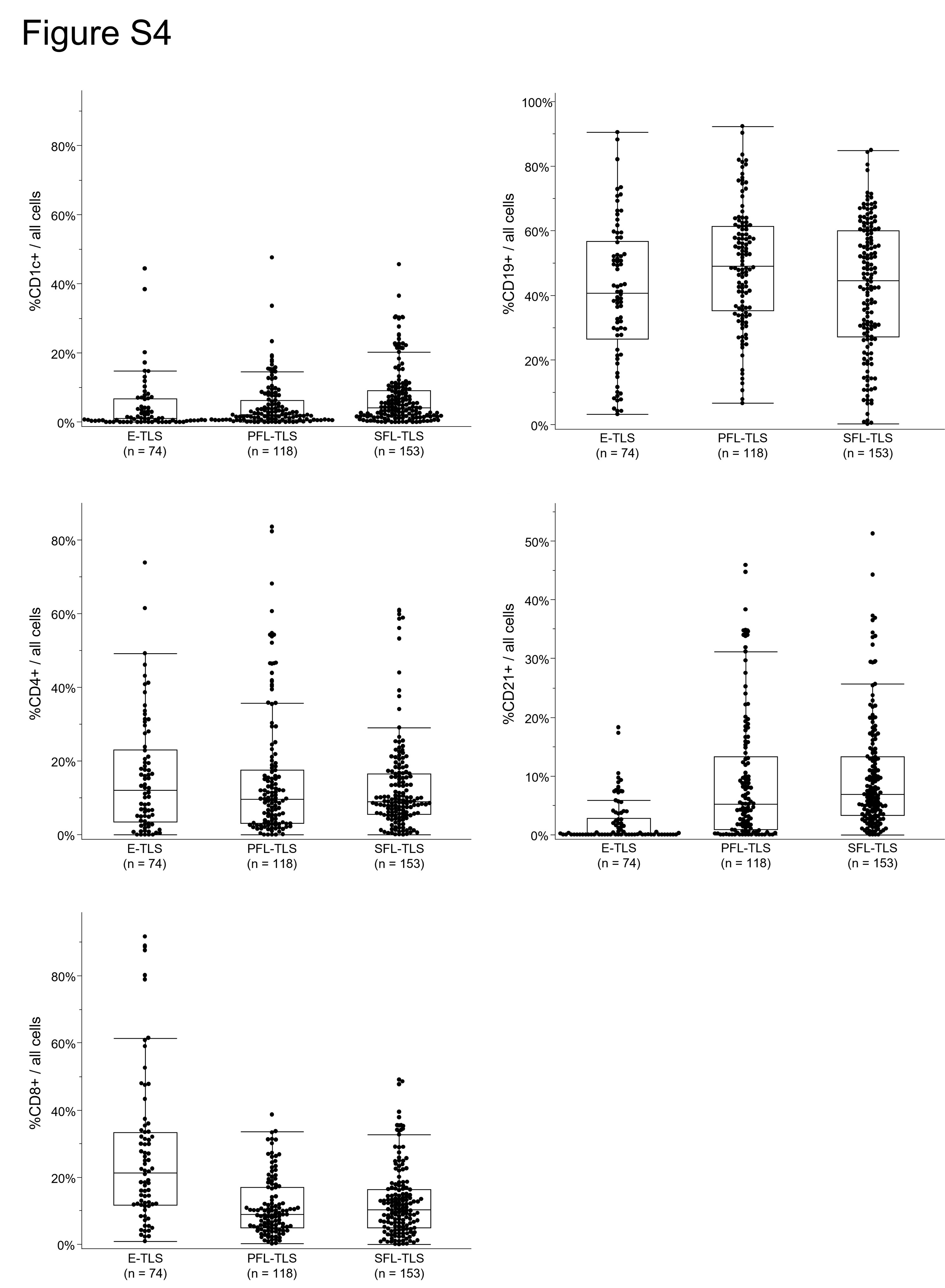

Supplement: Supplementary file 10 — Supplementary Figure S4 [file 41416_2023_2235_MOESM10_ESM.jpg]

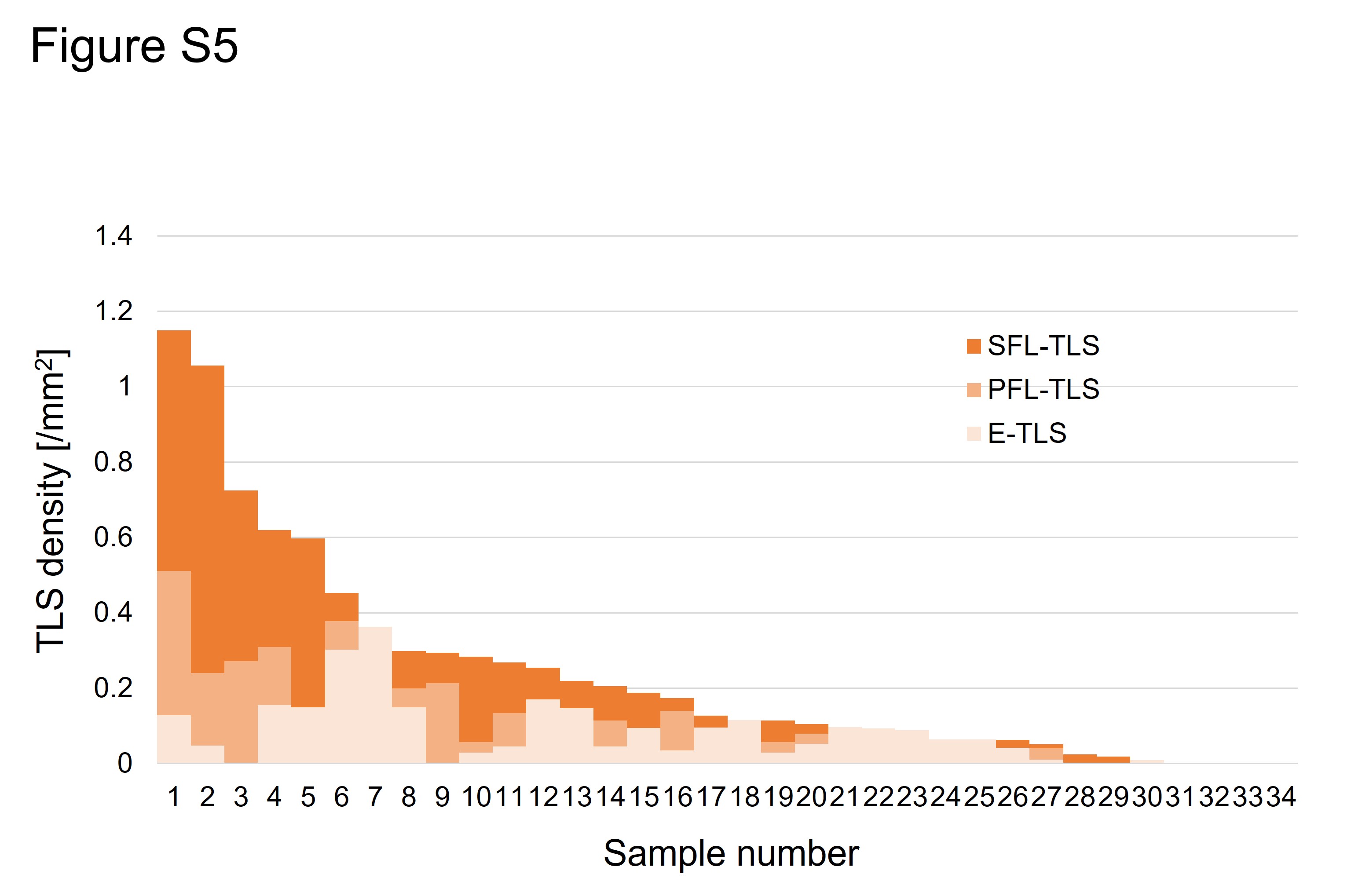

Supplement: Supplementary file 11 — Supplementary Figure S5 [file 41416_2023_2235_MOESM11_ESM.jpg]

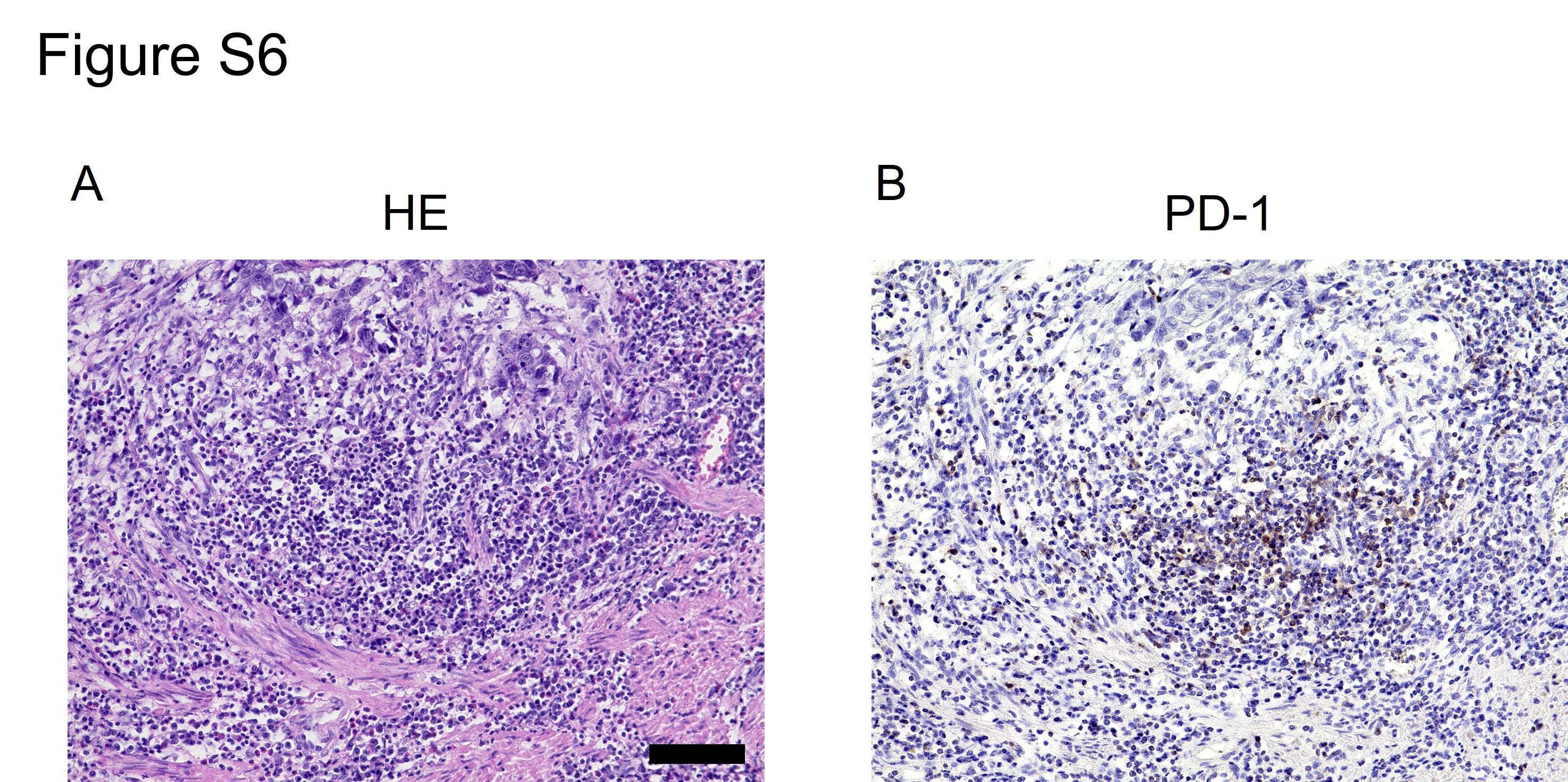

Supplement: Supplementary file 12 — Supplementary Figure S6 [file 41416_2023_2235_MOESM12_ESM.jpg]

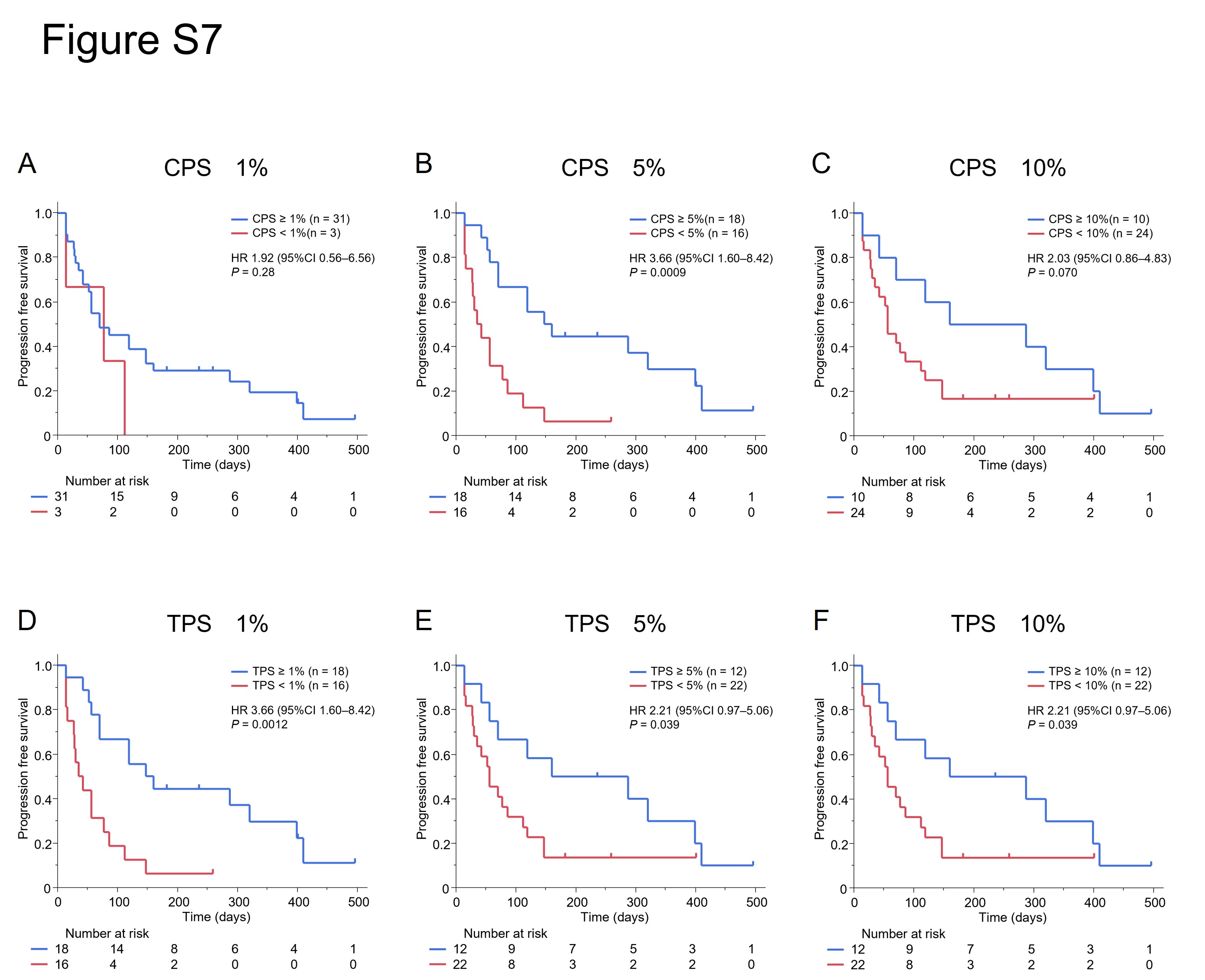

Supplement: Supplementary file 13 — Supplementary Figure S7 [file 41416_2023_2235_MOESM13_ESM.jpg]
